# Reverse Takotsubo Cardiomyopathy: A Case Report

**DOI:** 10.7759/cureus.107417

**Published:** 2026-04-20

**Authors:** Abigayle Wyer, Lucas N Canaan, Samuel Berding, Timothy J Stevens, Michael Cormican

**Affiliations:** 1 Surgery, Northeast Georgia Medical Center Gainesville, Gainesville, USA; 2 General Surgery, Northeast Georgia Medical Center Gainesville, Gainesville, USA; 3 Trauma and Acute Care Surgery, Northeast Georgia Medical Center Gainesville, Gainesville, USA

**Keywords:** broken heart syndrome, cardiomyopathy, heart failure, reverse takotsubo cardiomyopathy, takotsubo, trauma

## Abstract

Takotsubo cardiomyopathy (TCM), also known as “broken heart syndrome,” is classically characterized by apical akinesis with preserved basal contractility. A less common variant, termed "reverse TCM," demonstrates the opposite pattern, with basal akinesis and preserved apical function. We present the case of a 25-year-old female who developed acute heart failure with reduced ejection fraction following a ground-level fall resulting in a subdural hematoma (SDH). Subsequent evaluation revealed findings consistent with reverse TCM. This report discusses the clinical presentation, hospital course, and relevant literature surrounding this rare entity.

## Introduction

Takotsubo cardiomyopathy (TCM) has had multiple names over the years, including “broken heart syndrome," “stress-induced cardiomyopathy,” and “apical ballooning syndrome” [1]. The general definition of cardiac abnormality is akinesis of the cardiac apex with normal wall motion [1]. The predominant incidence of TCM is in patients with acute coronary syndrome at a rate of 1-2%, with the classical type of apical ballooning being the most common at approximately 80%.

Within the umbrella of TCM, there is a rare variant named reverse TCM, and that is the focus of this case report. The presentation of the reverse variant is normal to hyperkinesis of the cardiac apex with akinesis of the ventricular wall [2,3]. In the evaluation of the literature, among a study population of approximately 1,750 patients diagnosed with TCM, 2.2% had the reverse variant [2].

This case report details the workup and management of a 25-year-old female who developed acute heart failure with the finding of reverse TCM after presenting to a community level 1 trauma center after a ground-level fall.

## Case presentation

A 25-year-old female presented to the emergency department by emergency medical services (EMS) after a witnessed syncopal fall. She reportedly fell to her knees and then fell over, striking her head in a parking lot. EMS reported a GCS of 5 on scene, and she was intubated in the field. She presented to the trauma bay intubated and hemodynamically stable. Trauma imaging was notable for a 5.5 mm subdural hematoma (SDH) with a 6 mm midline shift. This finding prompted an emergent consultation to neurosurgery, and the patient was taken to the operating room for a craniotomy. In addition, her high-sensitivity troponin level was elevated to 314 and on repeat was 14,509. Her electrocardiogram (EKG) revealed sinus tachycardia with no ST-segment changes, and QTc was 450. She was admitted to the intensive care unit (ICU) postoperatively and was hemodynamically unstable, requiring pressor support.

**Figure 1:**
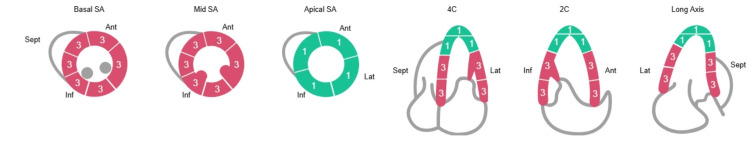
Diagram from the second transthoracic echocardiogram (TTE) that demonstrates akinesis (red) of the base of the left ventricle with hyperkinesis (green) of the apex of the left ventricle.

**Figure 2:**
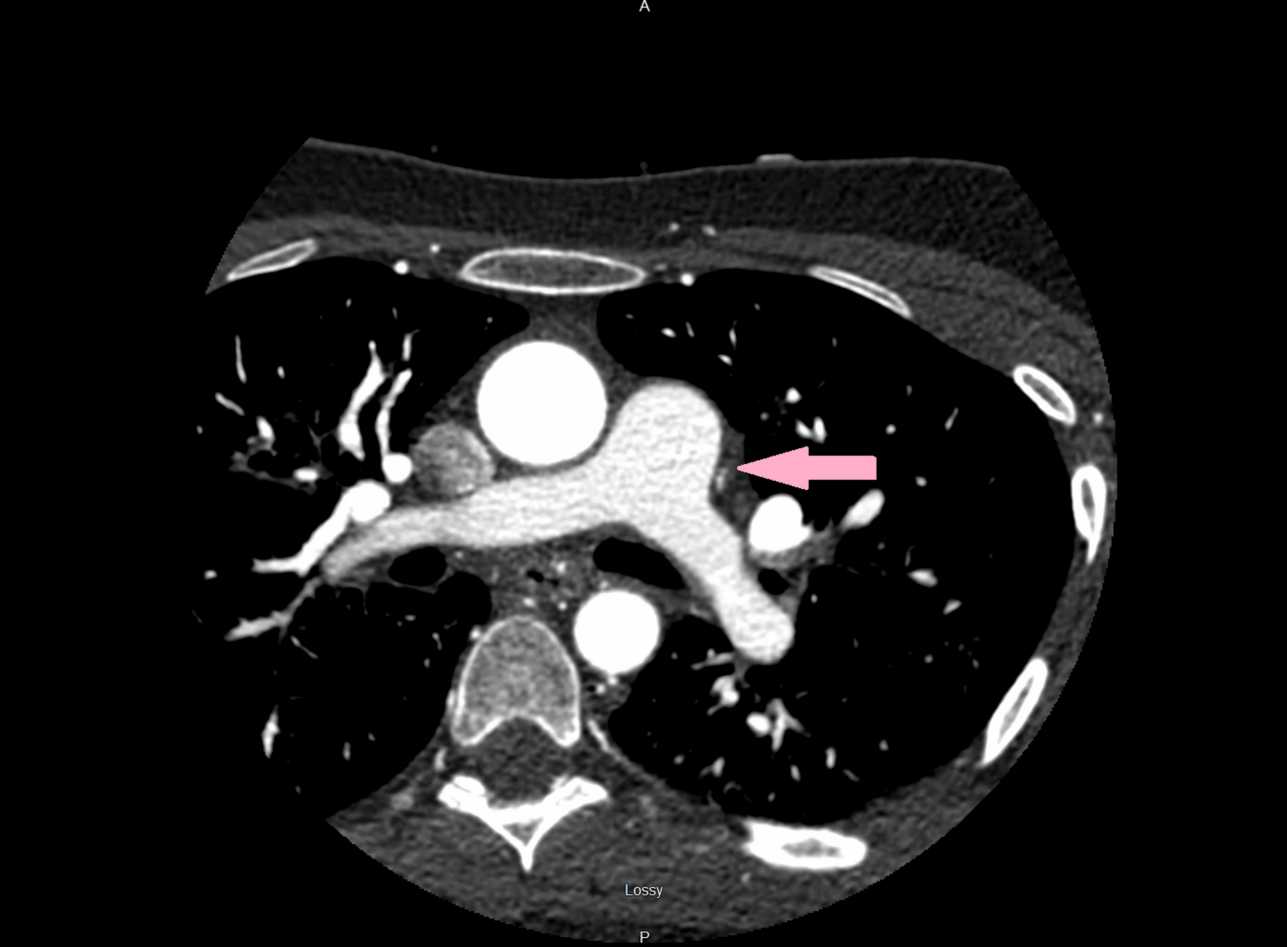
Computed tomography pulmonary angiogram (CTPA) revealing patent pulmonary artery with no signs of pulmonary embolism (pink arrow).

Postoperatively, her syncope evaluation was continued in the ICU. She underwent a transthoracic echocardiogram (TTE) that revealed a moderately reduced ejection fraction (EF) of 35-40%, notably with hypokinesis of the base of the left ventricle and normal motion of the apex of the heart. She was started on milrinone and responded well with normalizing blood pressure. During her ICU course, she continued to have worsening hypoxic episodes, and milrinone could not be weaned. She underwent a repeat TTE 24 hours later, and her EF reduced further to 20-25% with akinesis of the base of the left ventricle and hyperkinetic motion of the heart apex (Video 1, Figure 1). For the next few days, she continued to require vasopressor, inotropic, and ventilator support. On day 4 of her admission, she underwent repeat TTE, which revealed mild improvement in her EF to 30-35% and redemonstration of hypokinesis of the left ventricle base and hyperkinesis of the apex. Further workup included a CT angiogram for evaluation of a pulmonary embolism, which was negative (Figure 2).

**Video 1:** Transthoracic echocardiogram (TTE) demonstrating contraction of the apex of the left ventricle and akinesis of the base of the heart.

**Figure 3:**
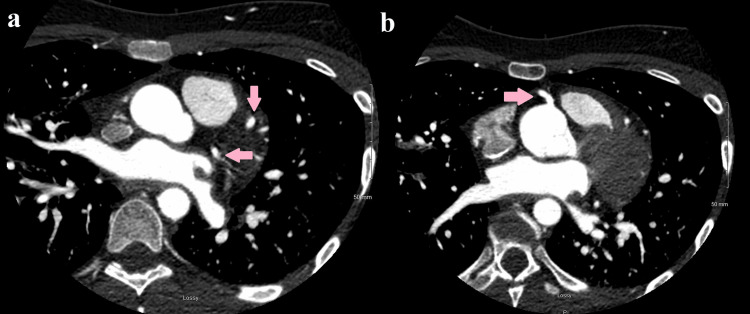
(a) Coronary computed tomography angiogram (CTA) demonstrating patent left-sided coronary arteries (pink arrows). (b) Coronary CTA demonstrating a patent right coronary artery (pink arrow).

On day 6 of her admission, she was extubated. On the following day, she was weaned off vasopressor and inotrope support. She ultimately improved from her cardiomyopathy and resumed ambulation by day 8 of her admission. She underwent a coronary CT angiogram to complete her cardiac workup, with no abnormal findings noted (Figure 3). She continued to improve back to her baseline of health, and she was discharged home on day 10.

She has been recovering well at home since discharge. She had a follow-up TTE about three months after discharge, which revealed complete resolution of cardiomyopathy findings (Video 2). 

**Video 2:** Follow-up three-month transthoracic echocardiogram (TTE) demonstrating complete resolution of previous acute cardiomyopathy findings with full contraction of apex and base of the heart.

The findings on her TTEs, including reduced EF with hypokinesis or akinesis of the base of the heart and normal to hyperkinetic motion of the apex of the heart, are classic findings for reverse Takotsubo syndrome or inverted TCM. The cause of her case of this syndrome is likely due to her traumatic brain injury, brain surgery, prolonged decompensated clinical status, or a combination.

## Discussion

TCM was first described in Japan in the 1980s and is typically associated with significant physical or emotional stress, earning the name “broken heart syndrome” [1]. It is characterized by transient akinesis of the apical myocardium with preserved or hyperkinetic contractility in the basal segments [4]. Although TCM remains an uncommon cardiac pathology, the reverse variant, characterized by basal akinesis with apical hyperkinesis, is even rarer.

The underlying mechanism of TCM is thought to involve catecholamine excess during periods of acute stress, leading to myocardial stunning from adrenergic overstimulation [5]. This abnormal cardiac response has been described following a variety of stressors, including trauma, acute coronary syndromes [5], and perioperative events such as liver transplantation [6].

The exact pathophysiology of both classic and reverse TCM remains incompletely understood. The predominant theories center on catecholamine-mediated myocardial toxicity and microvascular dysfunction [4,6]. Coronary vasospasm has also been implicated, with angiographic evidence of diffuse coronary spasm [4]. Alternatively, direct catecholamine toxicity may overwhelm local adrenergic receptors, leading to transient myocardial dysfunction and reduced contractility [7].

In this patient, echocardiography demonstrated basal akinesis with apical hyperkinesis, findings consistent with previously described cases of reverse TCM. The patient’s demographics also align with the population most affected, as this condition tends to occur more frequently in younger female patients [8,9]. Moreover, reverse TCM is often associated with acute neurological insults such as intracranial hemorrhage, which was the precipitating factor in this case [10].

TCM has been reported as a complication of aneurysmal subarachnoid hemorrhage in the literature [11]. This case is a unique variation from the current reports, given that the intracranial hemorrhage in this case is a traumatic subdural hemorrhage. In addition, this patient suffered from reverse TCM as opposed to traditional TCM. 

Syncope is defined as a transient loss of consciousness in addition to loss of posture or falling down [12]. Syncope can be caused by a number of different events. This patient likely experienced neurocardiogenic syncope given the TTE findings during admission. Neurocardiogenic syncope is due to an abnormal autonomic response that is poorly understood [12]. However, the current theory is cardiac C fiber activation leading to changes in heart rate or peripheral vascular tone, resulting in syncope and collapse [12].

There is no standardized treatment algorithm for TCM beyond supportive care [13]. Prognosis is generally favorable compared to other forms of stress-induced cardiomyopathy [14]. In this case, the patient required temporary inotropic and vasopressor support, which was successfully weaned as cardiac function recovered, consistent with prior case reports and existing literature on reverse TCM.

## Conclusions

This case demonstrates a classical presentation of a rare form of cardiomyopathy. First, the patient is young and suffered a traumatic event that led to acute heart failure. Second, the echocardiogram findings of acutely reduced ejection fraction with hypokinesis or akinesis of the base of the heart with preserved apical motion of the heart. Third, the patient required support through the decompensation but recovered fully within a couple of weeks. Bringing awareness to the unique form of cardiomyopathy is essential for prompt diagnosis and proper management of this rare syndrome.
